# Soil Aggregates and Fertilizer Treatments Drive Bacterial Interactions via Interspecies Niche Overlap

**DOI:** 10.1128/spectrum.02524-21

**Published:** 2022-03-02

**Authors:** Xiang Xiong, Hao Liao, Yanfang Xing, Xukun Han, Wanle Wang, Wenjie Wan, Qiaoyun Huang, Wenli Chen

**Affiliations:** a State Key Laboratory of Agricultural Microbiology, Huazhong Agricultural University, Wuhan, China; b Key Laboratory of Arable Land Conservation (Middle and Lower Reaches of Yangtze River), Ministry of Agriculture and Rural Affairs, College of Resources and Environment, Huazhong Agricultural University, Wuhan, China; Institut Pasteur

**Keywords:** bacterial relationship, connectance, interaction network, nestedness, niche overlap

## Abstract

Bacterial interactions play significant roles in ecological functions in responding to anthropogenic interference and soil structure changes. However, it remains largely unknown how fertilizer regimes and soil particle sizes drive bacterial interactions. To evaluate bacterial interaction patterns in soil aggregates under long-term fertilizer treatments, we sampled nine bacterial co-occurrence communities and compared the difference between interspecies resource consumption patterns and network structure. Despite the differences between fertilizer treatments, the negative correlation ratios of interaction networks in soil aggregates were macroaggregates > microaggregates > silt + clays. Likewise, NPK-supplement (chemical fertilizer) had also decreased the number of positive correlations of the interaction network than M-supplement (organic fertilizer), regardless of the size of soil aggregates. Linear model analysis revealed that interspecies trophic patterns, including niche overlap and nestedness, drove bacterial competition in the interaction networks. Most importantly, interspecies niche overlap may be the intrinsic factor in the effects of fertilizer treatments and soil aggregates on bacterial interactions. This study enhances our understanding of the potential for changes in species trophic patterns and might guide the promotion of land management.

**IMPORTANCE** Despite that the influence of soil structure and fertilizer treatments on the bacterial community has been widely studied, how they drive interspecies interactions has not been largely explored. Connectance and nestedness were significantly correlated with bacterial interactions, but no differences were found in different soil aggregates and fertilizer treatments. However, interspecies niche overlap could respond to soil aggregates and fertilizer treatments and ultimately drive the bacterial interactions. This study enhances our understanding of the mechanism of microbial interactions and highlights the importance of trophic patterns in the bacterial community. Our findings extend knowledge for nutrient availability on interspecific interactions.

## INTRODUCTION

Interspecies interactions in multispecies biofilms are essential for the sustainability and development of complex communities and significantly affect the function of an ecosystem. Nutrient interactions, such as cross-feeding or competition for niche space, are usually the dominant forms of bacterial interactions and ultimately determine bacterial co-occurrence (1). The empirical research found agricultural management and soil heterogeneity can remarkably affect the soil nutrition conditions and thus change the bacterial distribution and relationships (2, 3). However, the substantial variability in resource utilization patterns between species appears to be an intrinsic but poorly understood feature of bacterial interactions in the face of different environments.

Several mechanisms contribute to the bacterial interactions, including warfare (4), the temporal and spatial structure of the environment (5), dispersal and range expansion (6), and resources (7). With respect to the resource, three different trophic patterns, including niche overlap, nestedness, and connectance, are used to describe the resource interactions within bacterial communities. The interspecies connectance of nutritional resources is critical to the inhabitants of multispecies biofilms. Some members may be altruistic and secrete “public goods” to benefit other cells in the population of multispecies biofilms, so high connectance could efficiently exploit most resources (8, 9). In general, nestedness represents the trophic interactions among generalists and specialists in the interaction network and is important in the stability and diversity of the community. High nestedness could improve the survival of generalists growing on the downstream metabolic pathways due to their favorite substrates produced by some specialists (10). Meanwhile, nutrient consumption patterns are different among the specialists and generalists, which could alleviate their competition for niches and enhance the robustness of the community. However, high interspecies niche overlap would decrease the stability of the bacterial community due to the ecological similarity that leads to enhanced competition (11). Given the diversity of bacterial resource consumption characteristics in multispecies biofilms, which may ultimately lead to different interaction patterns, an investigation into the mechanisms of trophic patterns toward interspecies interactions seems warranted.

Soil contains large amounts of bacteria, which often exist as polymicrobial aggregates called multispecies biofilms (12). Soil aggregates are the fundamental unit of soil and have crucial effects on the abundance and diversity of microbial communities and even on some functional groups. In the black soils of northeastern China, the abundance of ureolytic microbes is higher in microaggregates than macroaggregates and silt + clays fractions (13), while the abundance of nitrite-oxidizing bacteria is higher in macroaggregates than for other particle sizes (14). Macroaggregates often contain higher labile soil organic carbon (SOC) contents (15, 16), while the SOC that is associated with microaggregates may be more biochemically recalcitrant (17). Bacteria are susceptible to changes in the soil environment, and even small changes can cause strong quorum behavior. Differences in nutrient content in soil aggregates may eventually lead to changes in species interactions and multispecies biofilms formation. Agricultural land management methods, such as fertilizer treatments, tillage, and land use, have a noticeable influence on various ecological processes and soil microbial communities (18–20). The impacts of fertilizer treatments on microbial diversity have been well studied (21, 22). Short-term organic fertilizer treatment increased the bacterial richness significantly, especially for some functional bacteria in Karst areas, such as *Nitrospira* and *Gemmatimonas* (23). Large amounts of chemical fertilizers have been applied to arable fields over the past few decades to prevent food shortages worldwide and maximize crop yields. Long-term chemical fertilizers may cause soil degradation and decrease bacterial diversity (24). Mitigation of soil properties is mediated mainly through the activities of soil-dwelling microorganisms and their interactions. The apparent differences in microbial diversity under agricultural management and soil structure changes enable the study of interspecific interactions response to environmental disturbances.

Each soil aggregate may form a unique evolutionary incubator to provide a characteristic environment for microbial communities and influence their coexistence (25). As a major agricultural management method, fertilizer treatments have essential effects on soil properties and bacterial communities. Both fertilizer treatments and soil aggregates could alter the diversity and composition of the bacterial community, but how they affect bacterial interactions is unclear. We hypothesized that the impact of fertilizer treatments and soil aggregates on bacterial interactions might include an indirect influence in which fertilizer treatments and soil aggregates first affect trophic patterns among species, and these trophic patterns then affect bacterial interactions, which eventually alter bacterial community structures. This study directly examined the distribution of trophic patterns in interaction networks as an underlying mechanism to predict bacterial interactions in response to environmental disturbances (e.g., soil aggregates and fertilizer treatments).

## RESULTS

### The isolates coculture from fertilizer treatments and soil aggregates.

**FIG 1:**
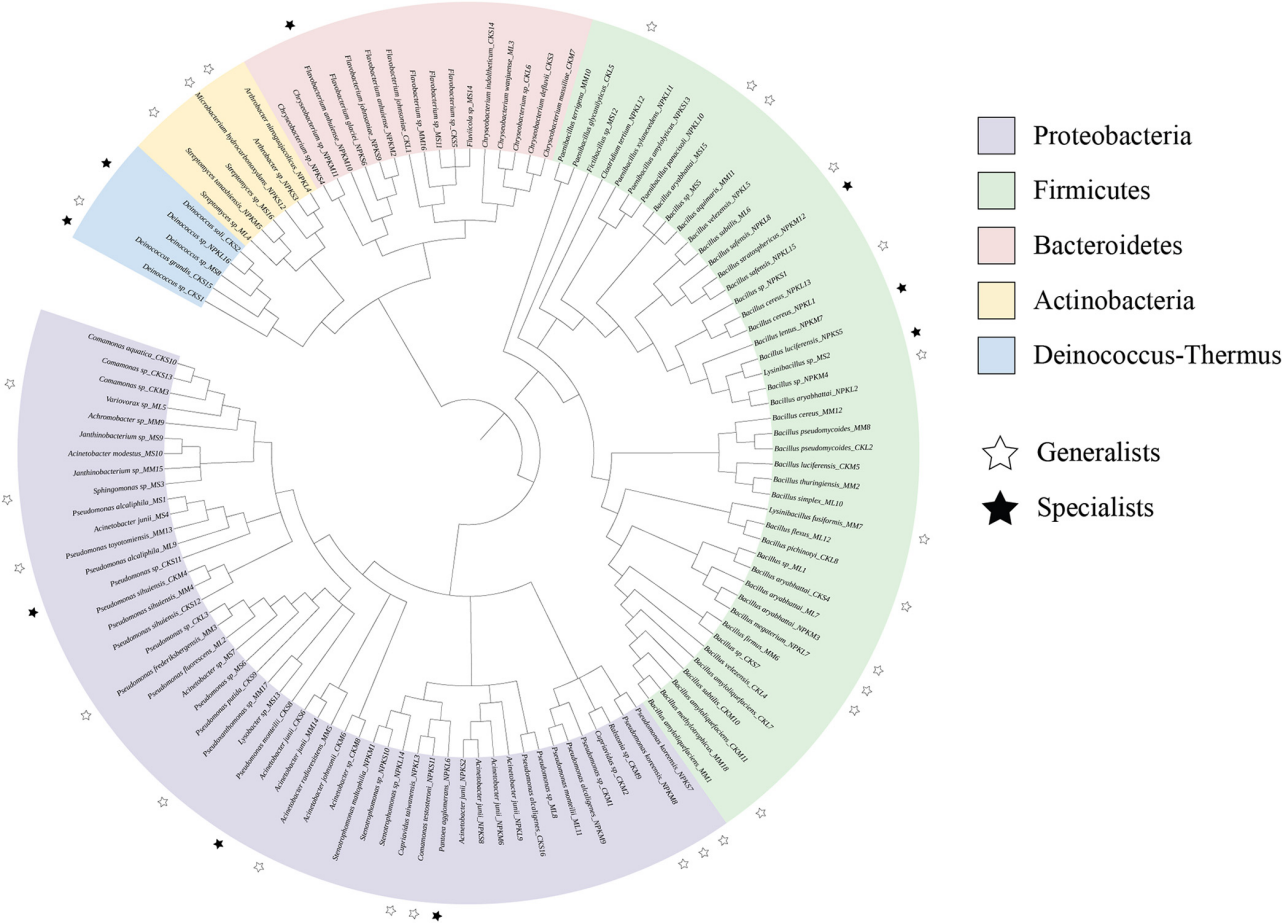
Phylogenetic tree of 122 bacteria isolated from the soil environment. The black stars represent the specialists, and the empty stars represent the generalists.

Based on the 16S rRNA gene sequencing analysis, 122 bacterial strains were isolated. These bacterial strains were classified into five phyla: Proteobacteria, Firmicutes, Bacteroidetes, Actinobacteria, and Deinococcus-Thermus. The bacterial resource consumption characteristics identified 9 specialists and 27 generalists (Fig. 1). Coculturing all possible combinations examined the prevalence of interspecific interactions among isolates under each fertilizer-treated soil. As shown in Table 1, 2450 different biofilm consortiums were screened. In the control without fertilizers (CK) treatment, the number of positive correlation pairs of species was 227, negative correlation pairs were 267, and the other 101 correlation pairs was the “unresolved” regime; In the swine manure (M)-supplement, the number of positive correlation pairs of species was 453, negative correlation pairs were 387, and the other 195 correlation pairs was the “unresolved” regime. In the nitrogen, phosphate, and potassium (NPK)-supplement, the number of positive correlation pairs of species was 183, negative correlation pairs were 503, and the other 134 correlation pairs were the “unresolved” regime.

**TABLE 1:** The interspecies interactions in different fertilizer treatments

Treatments	Positive	Negative	Unresolved
CK	227	267	101
M	453	387	195
NPK	183	503	134
Total			2450

### Interspecific trophic patterns in fertilizer treatments and soil aggregates.

**FIG 2:**
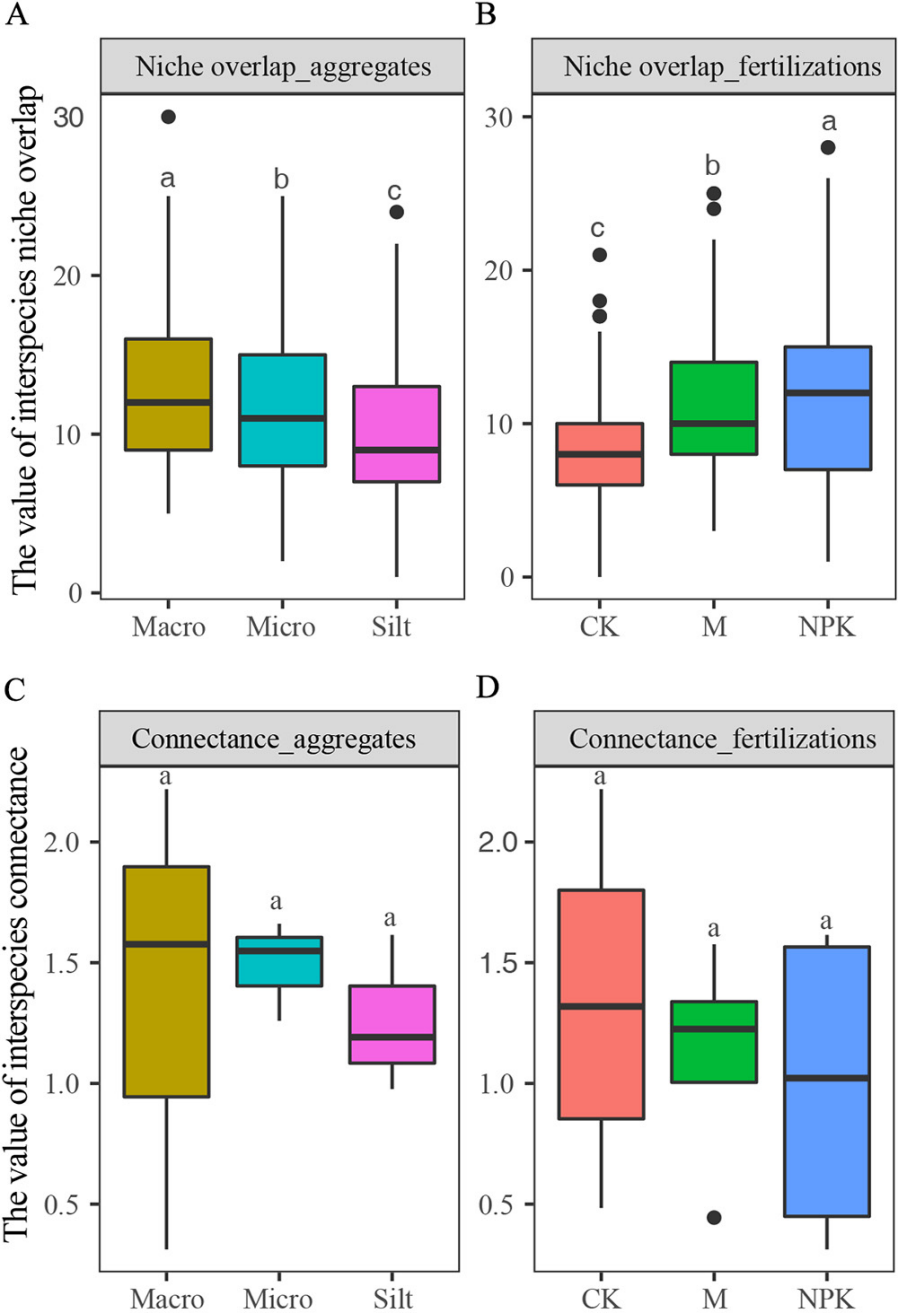
The distribution of interspecies trophic patterns (niche overlap and connectance) in fertilizer regime and soil aggregates. Macro, micro, and silt represent the macroaggregates, microaggregates, and silt + clays, respectively. Boxplot (A-D) with different lowercase letters indicate significant differences (*P* < 0.05) across soil aggregates, as revealed by one-way ANOVA with Turkey’s post hoc test.

To assess the influence of fertilizer treatments and soil aggregates on interspecies trophic patterns, we calculated the distribution of interspecific connectance and niche overlap in each soil sample. Fertilizer treatments significantly increased interspecific niche overlap, especially the NPK-supplement (*P* < 0.001). Meanwhile, niche overlap decreased with the decrease of soil particle size (*P* < 0.001). However, there was no significant difference in connectance between fertilizer treatments and soil aggregates (Fig. 2).

### Analysis of the roles of fertilizer treatments and soil aggregates on the interaction networks.

**FIG 3:**
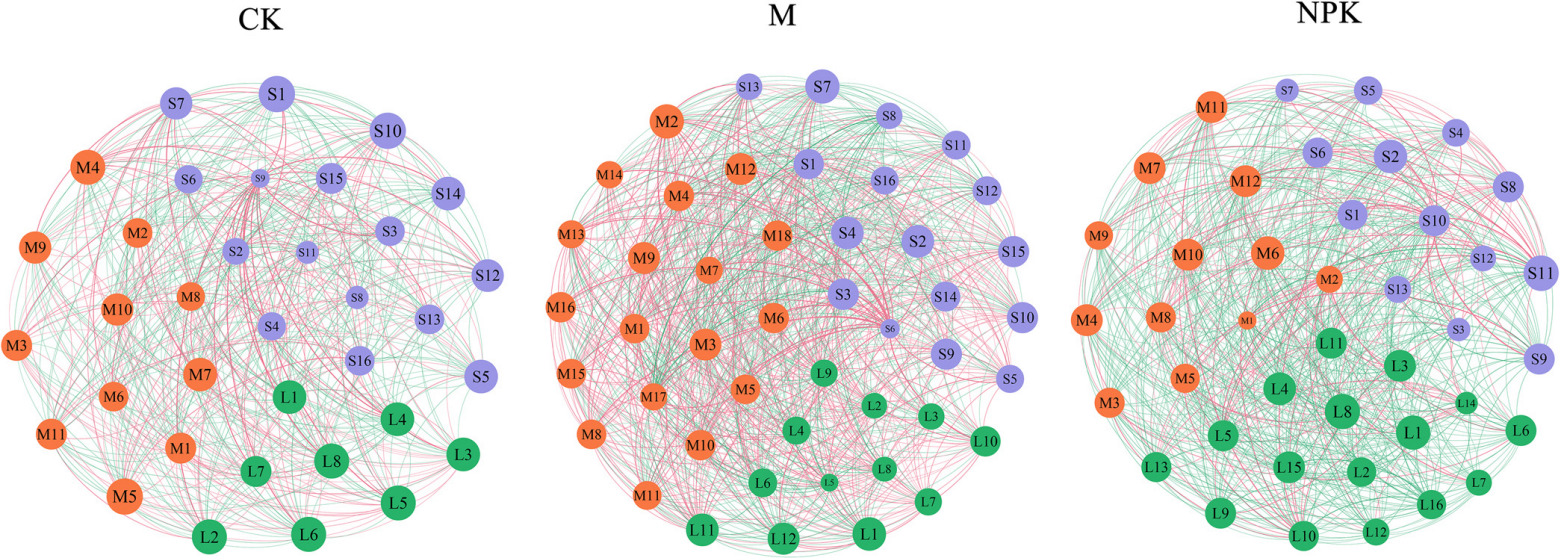
The network diagram of bacterial interactions under three fertilizer treatments (CK, M, NPK). The red lines mean the positive correlations and green lines mean the negative correlations in the network. The thickness of lines indicates the intensity of bacterial interaction. Each interaction network contains three soil particles sizes (macroaggregates, microaggregates, and silt + clays), and green nodes represent the bacteria isolated from the macroaggregates, orange nodes represent the bacteria isolated from the microaggregates, and purple nodes represent the bacteria isolated from silt + clays.

Using the weighted relationships of interspecies, we constructed interaction networks for the bacterial community from each fertilizer treatment and soil aggregates (Fig. 3) and detailed all pairwise interactions between isolates. The network properties are shown in Table 2. The negative correlation in NPK-supplement (73.32%) was higher than in CK treatments (54.05%) and M-supplement (46.07%) irrespective of the soil aggregate classification. Bacterial interactions in soil aggregate presented diversity between fertilizer and no-fertilizer treatments (Fig. S1). In the M-supplement and NPK-supplement treatments, the negative correlation was higher in the macroaggregates than microaggregates and silt + clays and followed by the M-supplement groups, where macroaggregates (38.78%) > microaggregates (29.47%) > silt + clays (26.4%), and the NPK-supplement groups, where macroaggregates (84.91%) > microaggregates (53.57%) > silt + clays (41.27%). However, the negative correlation was higher in the silt + clays (60.67%) followed by the macroaggregates (43.48%) and microaggregates (30.61%) the CK treatment.

**TABLE 2:** The topological properties of the interaction networks

Sample	Nodes	Edges	Positive (%)	Negative (%)	Avg degree	Avg weighted degree	Modularity	Avg clustering coefficient	Avg path length
CK	35	494	45.95	54.05	28.229	66.692	0.117	0.841	1.17
CK_macroaggregates	8	23	56.52	43.48	5.75	7.123	0.05	0.836	1.179
CK_microaggregates	11	49	69.39	30.61	8.909	17.011	0.04	0.878	1.109
CK_silt+clays	16	89	39.33	60.67	11.125	33.74	0.166	0.792	1.258
M	46	840	53.93	46.07	36.522	67.542	0.092	0.823	1.188
M_macroaggregates	12	49	61.22	38.78	8.167	17.828	0.091	0.764	1.258
M_microaggregates	18	125	73.6	26.4	13.889	30.977	0.1	0.819	1.183
M_silt+clays	16	95	70.53	29.47	11.875	21.958	0.122	0.844	1.208
NPK	41	686	26.68	73.32	33.463	48.582	0.114	0.857	1.163
NPK_macroaggregates	16	106	15.09	84.91	13.25	16.252	0.113	0.879	1.117
NPK_microaggregates	12	56	46.43	53.57	9.333	12.776	0.081	0.86	1.152
NPK_silt+clays	13	63	58.73	41.27	9.692	24.571	0.019	0.834	1.192

### The correlations between the bacterial relationships and interspecific trophic patterns.

**FIG 4:**
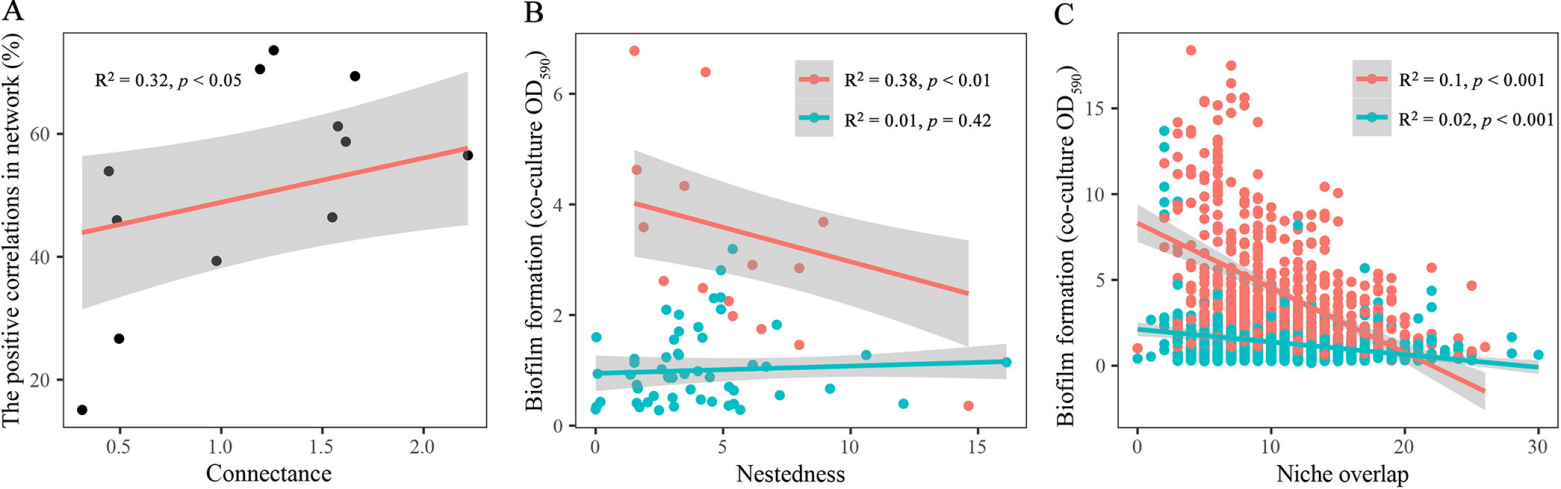
The correlation between the trophic patterns with bacterial interactions. (A) The linear models of interspecies connections and percentage of positive correlations in networks, A total of 12 connectances were obtained (12 networks). (B) The relationship between the interspecies nestedness and the intensity of bacterial interactions. (C) The relationship between the interspecies niche overlap and the intensity of bacterial interactions. The red points mean the value of the positive correlation and the green points mean the value of negative correlations.

We first assessed the correlations between connectance and positive relationships based on the interaction networks. It indicated that connectance was significant to promote the positive correlation of the bacterial community (Fig. 4A; R^2^ = 0.33, *P* < 0.05). Likewise, the intensity of positive correlation was significantly decreased with the niche overlap and nestedness (niche overlap: R^2^ = 0.1, *P* < 0.001; nestedness: R^2^ = 0.38, *P* < 0.01). However, the intensity of negative correlation was only correlated with the niche overlap (R^2^ = 0.02, *P* < 0.001; Fig. 4B to C).

## DISCUSSION

While fertilizer regimes and soil particles sizes have been shown to have diverse effects on microbial communities, it has not yet been addressed whether these effects permeate into the characteristics of naturally assembled microbial communities. Fertilizer treatments increased niche overlap, suggesting that nutrient inputs significantly change microbial communities’ resources consumption characteristics of microbial communities. It is reasonable that adding exogenous substances could increase the diversity of resources, while this type of agricultural management also increases the competition of bacteria for similar resources (26). At the same time, the synergistic consumption of complex organic matter by microorganisms may alleviate the competition for resources to some extent, which may be the reason for the highest niche overlap after the application of NPK-supplement than M-supplement, at least among culturable bacteria (27). The lower niche overlap of silt + clays suggests that spatial pressures selectively screen species with low niche overlap, allowing them to coexist (25). When bacteria are cocultured in an environment where long-term coexistence is impossible, bacterial competition will increase more significantly than in a sympatric environment (28). Application of organic fertilizer and chemical fertilizer can alter aggregate distributions and the organic carbon and nitrogen levels associated with soil aggregates (29). Sometimes, soil aggregates can also affect the distribution and interactions of microorganisms independently to a certain extent. Most importantly, contrary to the intuitive cognition that physicochemical properties are the mediate factor for bacterial distribution, the present data further showed that fertilizer treatments and soil aggregates could influence the interspecies trophic patterns (niche overlap), suggesting that this may be the internal element that the external environment impacts bacterial distribution.

We used network analyses to characterize the structure of bacterial interactions under distinct fertilizer treatments and found that NPK-supplement could significantly increase the negative correlations of interspecies compared to M-supplement and CK treatment. Similar results were found in previous studies, which detected a negative correlation of about 74% of bacteria in the chemical fertilizer sample based on the assessment of dual-biofilm yield (30). The reason may be that applying simple chemical fertilizer enhances the fierce bacterial competition for the directly available resources (27). Interestingly, no matter what fertilizers are applied, negative correlations decreased as the soil particle sizes decrease. It indicated that soil aggregates may serve as a relatively independent space and influence bacterial coexistence to some degree. The lower content of labile SOC in silt + clays might promote the niche expansion of the bacterial community and decrease the competition for similar resources (16, 17). These observations provide direct evidence that soil structure and fertilizer treatments could affect soil microbial interactions from the perspective of biofilms formation.

To understand the underlying mechanism that drives bacterial interactions, we focused on the resources’ links between species of the interaction networks. Trophic patterns of this type, which are represented by the configuration and distribution of different nutrients in the bacterial community, can provide strong predictions of the function and stability of ecosystems. The results indicated that niche overlap significantly reduced the intensity of interactions in both positive and negative correlation pairs. High niche overlap means high interspecies resource similarity, which leads to an increased degree of competition, and it could decrease the probability of bacterial coexistence or multispecies biofilms formation (31). Resident communities with high nestedness indicated intense competition among specialists and generalists for resources (32). However, interspecies nestedness was no different from the intensity of bacterial competition, and it was implied that the mechanism of multispecies biofilms formation was not only correlated with the resource but was also determined by the species composition of the bacterial community (33). The multispecies distribution in terms of its spatial organization would determine multispecies biofilms formation, and each species occupies a defined microsite to provide a balance among positive and negative interactions that enhance biofilms production (34). High connectance can decrease pathogen invasion due to the efficiency of exploiting most resources in the rhizosphere communities, but it can also increase members’ competition for resources (35). Our study indicated that high connectance could drive bacterial cooperation in the community. One possibility might be determined by spatially homogenous and heterogeneous. Bacteria could consume resources more efficiently in a spatially homogeneous microscopic environment, such as a liquid medium, than in a spatially heterogeneous, such as a soil environment (36, 37). Another possibility is that high connectivity promotes the consumption of complex organic matter by microbial communities through synergy and maintains the stability of the community (9). These results indicated that different network resource characteristics might shape species interactions in bacterial communities. However, there was no significant difference of connectance in different fertilizer treatments and soil aggregates, suggesting that niche overlap could respond to changes in fertilizer treatments and soil aggregates, which ultimately changed bacterial interactions. Finally, the NPK-supplement and macroaggregates could significantly increase the interspecies niche overlap, accounting for the high negative correlation in both environments. We suppose that the combined application of M-supplement and NPK-supplement could mitigate or even prevent the adverse effects of NPK-supplement alone on microbial networks. Therefore, we propose that M-supplement helps maintain the interaction networks of mainly positive correlations microorganisms.

In this study, we focused on interspecies trophic patterns, including niche overlap, connectance, and nestedness, one important means by which microbes can potentially interact with another within the community. The widespread differences between bacterial interaction networks were due to fertilizer treatments and soil aggregates that alter the interspecies trophic patterns, such as niche overlap. These results lay a foundation for further research on the nature and relevance between microbial interaction and bacterial community composition. Further research is needed to provide higher resolution natural assemblage microbial community analysis to determine the relationship between resource consumption characteristics of microorganisms in networks as a result and the cause of ecological function.

## MATERIALS AND METHODS

### Separation of soil aggregates and bacterial isolation.

The experimental soil site is located at the long-term fertilizer field of Laiyang (36°9′N, 120°7′E), Shandong Province, China. Moreover, the soil is a non-calcareous fluro-aquic soil (Alfisol, USDA soil taxonomy), and the site has a warm temperate semihumid monsoon climate with a mean annual temperature of 11.2°C and yearly rainfall of 779.1 mm. The fertilizer experiment has been implemented in a wheat-corn crop rotation system since 1978 and included three replicated plots with three treatments: control without fertilizers (CK); swine manure fertilizer (M); and chemical fertilizer (nitrogen, phosphate, and potassium fertilizers [NPK]). Urea was applied as a nitrogen (N) fertilizer at 276 kg×N/ha, superphosphate was applied as phosphate (P) fertilizer at 90 kg×P/ha and potassium chloride as potassium (K) fertilizer at 135 kg×K/ha. The swine manure fertilizer was provided at 60000 kg×N/ha, including 2 to 3 g/kg N, 20% to 50% organic matter, and 0.1 to 0.4 g/kg P (38).

Soil samples were collected at a depth of 0 to 20 cm in May 2017 and were placed in sterile plastic bags for transportation to the laboratory within 24 h after collection. Briefly, six soil subsamples (approximately 5 cm in diameter) were randomly selected from each experimental plot and then composited to gain one soil sample. Three aggregate-size classes were manually fractionated through wet sieving of 100 g of fresh soil on a series of three sieves (e.g., 2,000 μm, 250 μm, and 53 μm) as follows: macroaggregates (250 to 2,000 μm, L); microaggregates (53 to 250 μm, M) and silt + clays (<53 μm, S) (39).

Bacteria were isolated from soil aggregates using standard serial dilution plate techniques described in our previous study (33). Briefly, soil incubation solutions were prepared by making an initial 1:9 soil PBS mixture and the soil suspension was shaken at 180 rpm for 2 h to release the microbes. The soil suspension was then diluted to 10^−5^, and 150 μL suspension was plated in the in triplicates onto tryptic soy agar (TSA; tryptone, 15 g; soytone, 5 g; NaCl,5 g; Agar,15g; H_2_O, 1 L; pH, 7.4) and incubated at 28°C for 1 to 2 days. The cycloheximide (40 mg/liter) was added to inhibit the growth of fungi. Bacterial isolates were identified based on their phenotypes and sequencing of their 16S rRNA genes (27F: 5′-AGAGTTTGATCCTGGCTCAG-3′; 1492R: 5′-GGYTACCTTGTTACGACT T-3′) (40). The bacterial strains isolated in this study from the soil aggregates are listed in Table S1.

### Characterization of bacterial trophic competition patterns.

The resource consumption characteristics of bacteria were used to determine the indirect trophic patterns among species in the interaction networks. The survival of single species under 46 different single carbon resources was analyzed to determine the metabolic profile of the bacteria (Table S2). Briefly, exponential-phase cultures of the strains were pelleted by centrifugation (4,000 × *g*, 5 min); washed three times in 0.85% NaCl, and a mineral minimal medium that was supplemented with a 10 mM concentration of a single resource was used to adjust the optical density at 600 nm (OD_600_) to 0.1 (41). Finally, 200 μL was added to the 96-well tissue culture plate, shaking at 180 rpm and 28°C for 48 h. The wells with OD_600_ >0.05 were scored as exhibiting growth on the given substrate. We first estimated two resource consumption patterns: niche overlap and nestedness. Niche overlap was defined as the percentage of two species using the same carbon source in the metabolic profile (42). We describe as specialists who grew in less than 25% of the 46 single carbon resources and as generalists those isolates grown in more than 50% of all single carbon resources (10). Nestedness was computed by the R package bipartite using the nested function. Connectance was estimated using formula C = L/S^2^, where L was the sum of the number of carbon sources used by each species, and S denoted species richness of the interaction network (43).

### Bacterial coculture and assessment of the biofilms formation.

The strain consortia, originating from each fertilizer treatment (CK, M, NPK) were assessed for biofilms formation in monoculture and coculture. These strains were subcultured from frozen glycerol stocks into tryptic soy broth (TSB) medium (tryptone, 15 g; soytone, 5 g; NaCl, 5 g; H_2_O, 1 L; pH, 7.4) for 1 day at 28°C and then stocked onto TSA plates for 2 days at 28°C for the mono-colonies. Colonies from TSA plates were inoculated into 5 mL of TSB medium and were incubated under shaking (180 rpm) at 28°C overnight. Approximately 500 μL of these stationary-phases bacterial cultures were transferred to a fresh TSB medium and were grown until the exponential phase. The cell suspensions were adjusted to an optical density of 0.15 at 600 nm (OD_600_) in the TSB medium. A total of 160 μL of mono-species or two species (80 μL of each species) of exponentially growing cultures were added to the wells of 96-well tissue culture plates at 180 rpm at 28°C for 12 h. Biofilms assays were performed three times on different days with four technical replicates each time.

Quantification of biofilms was based on staining with crystal violet (CV) (44). The planktonic cells in each well were carefully removed with PBS. Then, 320 μL of 0.1% (wt/vol) CV was added, and the biofilms were stained for approximately 20 min followed by 360 μL of PBS to remove the redundant CV three times. The CV (dissolved in 96% ethanol) was measured in absorbance at 590 nm (A_590_) to represent the biofilm productivity. When the value of A_590_ exceeded 2, dilution with 96% ethanol was used to obtain the final biofilm content.

### Construction of the interspecies interaction networks.

**FIG 5:**
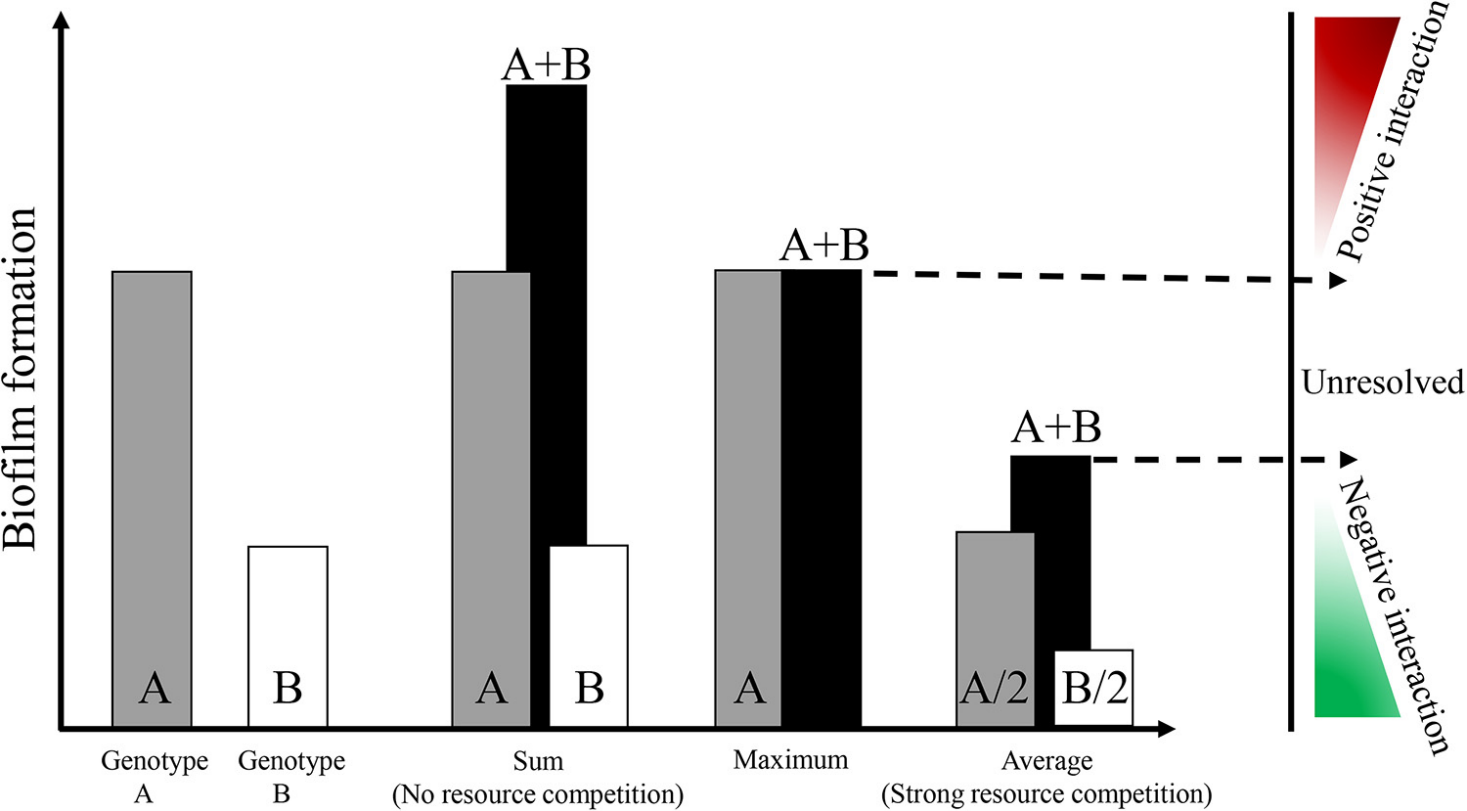
Classification scheme used for the assessment of bacterial relationships. Gray bars and white bars indicate monoculture biofilm formation by genotype A and B, respectively. Black bars are used to classify biofilms formation for coculture (left). Positive interaction is determined when the coculture produces more biofilm than the best monoculture biofilm producer. Negative interaction is determined when the coculture produces less than the averages of the monoculture.

Classifications of bacterial relationships in cocultures were performed by a hypothesis test approach about their biofilms yields (28). This hypothesis is dependent on a strict null model. Suppose there is no resource competition among the cocultured strains. In that case, the bacteria are expected to be equally productive in coculture as monocultures, and their biomass will be the same as the sum of the single cultured strains. However, these bacteria are isolated from the same environment and have similar resource preferences. Therefore, as shown in Fig. 5, biofilm synergy (positive correlation) occurs when more biofilms are formed from the coculture than from the best single biofilm. On the contrary, biofilm antagonistic (negative correlation) occurs when less biofilm is formed in cocultures than the average of monocultures. Meanwhile, the intensity of interactions was determined by the productivity of biofilms in coculture; the more biofilms produced by coculture, the stronger the synergistic effect, and vice versa. To ensure the stringency of the classification scheme, we also described the “unresolved” regime.

The interaction networks were constructed by the relationships of interspecies produced by the above process. The nodes of the network were culturable bacterial isolates; the weight of edges in the network was the yield of biofilms under the coculture of two bacteria. Therefore, three networks were created based on the fertilizer treatments (CK, M, NPK), and the edges in each network included the bacterial relationships within and between soil aggregates. Then nine subnetworks were created based on the bacterial relationships in each soil aggregates (macroaggregates, microaggregates, and silt + clays). All the networks were plotted using a Fruchterman-Reingold algorithm in Gephi (https://gephi.org/) (45). Meanwhile, the fundamental properties of interaction network structure, such as average degree, average weighted degree, average clustering coefficient, average path length, were calculated.

### Statistical analyses.

Unless otherwise stated, analyses were conducted in R version 4.1.0. The phylogenetic tree of bacterial isolates was constructed by the MEGA 7.0 and the classification of generalists and specialists were visualized with iTOL (https://itol.embl.de/). Three general linear models expressing biofilms formation in coculture were set up as a function of interspecies trophic patterns. One-way ANOVA was used to detect the significance of interspecies trophic patterns in the fertilizer treatments and soil aggregates. In this analysis, when we are concerned about fertilizer treatments, soil aggregates are not considered and classified into each fertilizer treatment, and vice versa.

### Data availability.

The 16S sequence for each bacterial isolate can be accessed through the NCBI SRA BioProject accession number PRJNA799129.
