# Supplementary material for: Soil Aggregates and Fertilizer Treatments Drive Bacterial Interactions via Interspecies Niche Overlap
Source: Microbiol Spectr. 2022 Mar 2;10(2):e02524-21. doi: 10.1128/spectrum.02524-21 (PMC8941866; doi:10.1128/spectrum.02524-21)
Supplement: Supplemental file 1 — Fig. S1; Tables S1 and S2. Download spectrum.02524-21-s0001.pdf, PDF file, 0.4 MB [file spectrum.02524-21-s0001.pdf]

Supplementary information

Soil aggregates and fertilization drive bacterial interactions via trophic patterns of interspecies

Xiang Xiong[1], Hao Liao[1], Yanfang Xing[1], Xukun Han[1], Wanle Wang[1], Wenjie Wan[1], Qiaoyun Huang[1,2], Wenli Chen[1*]

[1]State Key Laboratory of Agricultural Microbiology, Huazhong Agricultural University, Wuhan 430070, China

[2]Key Laboratory of Arable Land Conservation (Middle and Lower Reaches of Yangtze River), Ministry of Agriculture and Rural Affairs, College of Resources and Environment, Huazhong Agricultural University, Wuhan 430070, China

***Corresponding authors:**

Wenli Chen,

State Key Laboratory of Agricultural Microbiology, Huazhong Agricultural University, Wuhan 430070, China

wlchen@mail.hzau.edu.cn;

Phone: +86-27-87280670; Fax: +86-27-87280670

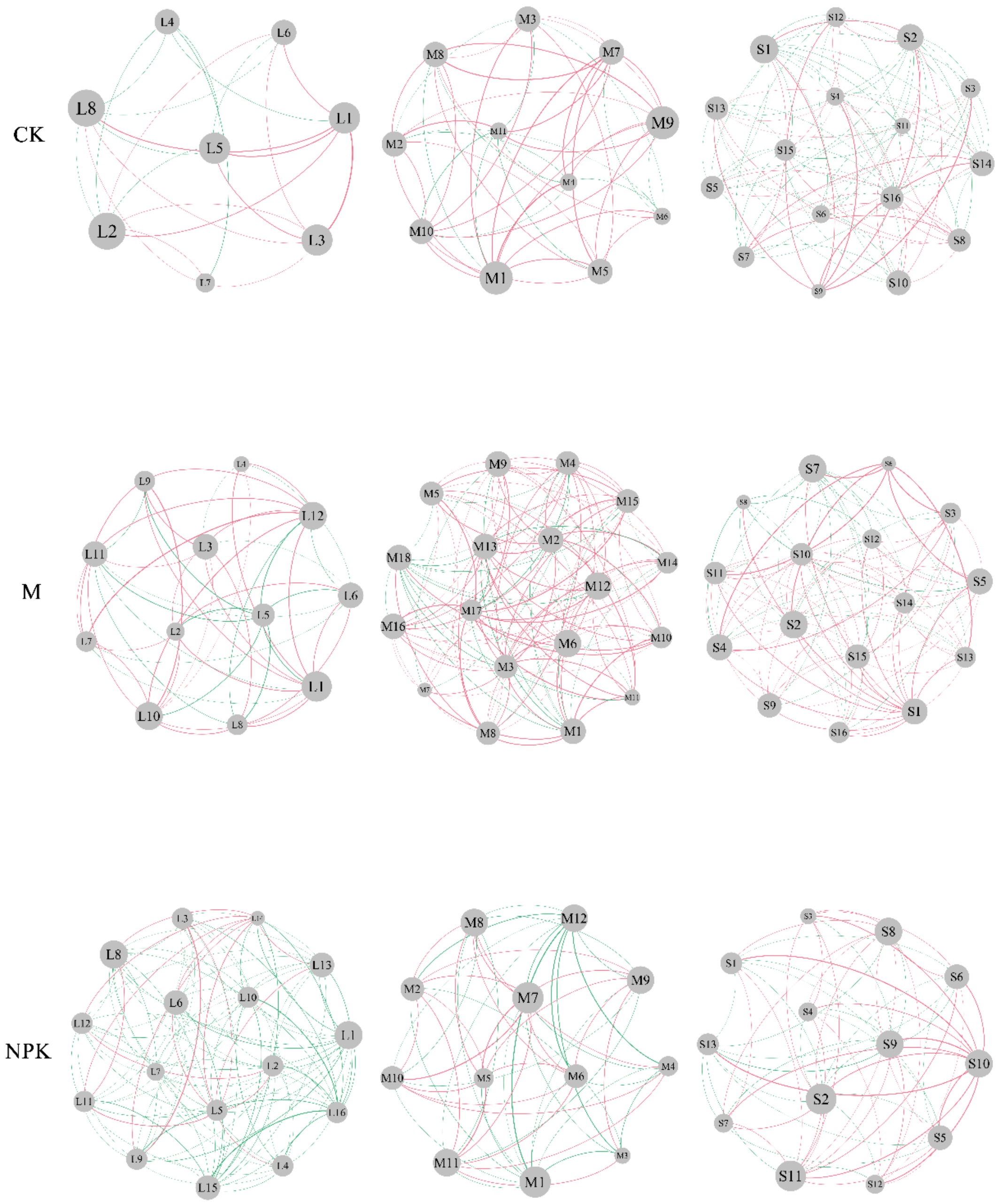

Fig. S1 The network diagram of bacterial interactions under soil aggregates (macro-aggregates, micro-aggregates, silt + clays). The red lines mean the positive correlation and green lines mean the negative correlation in the network. The thickness of lines indicates the intensity of bacterial interaction. “L” represent the bacteria isolated from the macro-aggregates, “M” represent the bacteria isolated from the micro-aggregates, and “S” represent the bacteria isolated from silt + clays.

Table S1 Organisms used in this study.

Species	*Closest match*	Similarity (%)	GenBank No.
CK-L1	*Flavobacterium johnsoniae*	97.44	KM252925
CK-L2	*Bacillus pseudomycoides*	96.88	MK373763
CK-L3	*Pseudomonas* sp.	99.58	KC236611
CK-L4	*Bacillus velezensis*	99.72	JN700101
CK-L5	*Paenibacillus glycanilyticus*	97.76	JF496313
CK-L6	*Chryseobacterium* sp.	97.63	JF700382
CK-L7	*Bacillus amyloliquefaciens*	99.41	KX980396
CK-L8	*Bacillus pichinotyi*	99.72	KC019189
CK-M1	*Pseudomonas* sp.	96.74	KP747656
CK-M2	*Cupriavidus* sp.	100	MF421555
CK-M3	*Comamonas* sp.	97.18	MG754434
CK-M4	*Pseudomonas sihuiensis*	100	AB681538
CK-M5	*Bacillus luciferensis*	99.72	MH497613
CK-M6	*Acinetobacter johnsonii*	97.3	MG594813
CK-M7	*Candidatus Chryseobacterium massiliae*	96.49	KP318469
CK-M8	*Acinetobacter* sp.	97.53	LC140820
CK-M9	*Ralstonia* sp.	100	DQ309421
CK-M10	*Bacillus subtilis*	100	MT110640
CK-M11	*Bacillus amyloliquefaciens*	99.72	KX871898
CK-S1	*Deinococcus* sp.	99.92	JQ511861
CK-S2	*Deinococcus soli*	96.28	CP011389
CK-S3	*Chryseobacterium defluvii*	97.66	KY194732
CK-S4	*Bacillus aryabhattai*	100	MT072179
CK-S5	*Flavobacterium* sp.	98.49	KF424280
CK-S6	*Acinetobacter junii*	100	MN581668
CK-S7	*Bacillus* sp.	99.21	KF366710
CK-S8	*Pseudomonas monteilii*	100	MN758773
CK-S9	*Pseudomonas putida*	100	MT020300
CK-S10	*Comamonas aquatica*	99.65	MF354014
CK-S11	*Pseudomonas* sp.	100	MN784298
CK-S12	*Pseudomonas sihuiensis*	97.52	LT629797
CK-S13	*Comamonas* sp.	97.83	MH890458
CK-S14	*Chryseobacterium indoltheticum*	98.76	CP033929
CK-S15	*Deinococcus grandis*	99.76	JN084140
CK-S16	*Pseudomonas alcaligenes*	100	KC764976
M-L1	*Bacillus* sp.	99.93	KJ504161
M-L2	*Pseudomonas fluorescens*	99.15	MF000304
M-L3	*Chryseobacterium wanjuense*	98.58	AB682410
M-L4	*Streptomyces* sp.	98.86	KP723794
M-L5	*Variovorax* sp.	99.35	KU821105
M-L6	*Bacillus subtilis*	100	KX901793
M-L7	*Bacillus aryabhattai*	99.93	KX230137

M-L8	*Pseudomonas* sp.	99.92	LC133613
M-L9	*Pseudomonas alcaliphila*	99.78	CP016162
M-L10	*Bacillus simplex*	98.85	KY316443
M-L11	*Pseudomonas monteilii*	100	JF681286
M-L12	*Bacillus flexus*	99.49	KJ719246
M-M1	*Bacillus amyloliquefaciens*	99.86	JF899255
M-M2	*Bacillus thuringiensis*	99.65	KJ831618
M-M3	*Pseudomonas frederiksbergensis*	99.79	KF424284
M-M4	*Pseudomonas sihuiensis*	99.37	LT629797
M-M5	*Acinetobacter radioresistens*	100	KX977571
M-M6	*Bacillus firmus*	99.79	JN210569
M-M7	*Lysinibacillus fusiformis*	99.79	KU179364
M-M8	*Bacillus pseudomycoides*	99.51	KJ767330
M-M9	*Achromobacter* sp.	99.77	KY435972
M-M10	*Paenibacillus terrigena*	98.81	GQ284528
M-M11	*Bacillus aquimaris*	99.86	KF933659
M-M12	*Bacillus cereus*	97.23	KY123862
M-M13	*Pseudomonas toyotomiensis*	100	KF010921
M-M14	*Acinetobacter junii*	100	CP024632
M-M15	*Janthinobacterium* sp.	97.6	HQ327125
M-M16	*Flavobacterium* sp.	99.36	AM922192
M-M17	*Pseudoxanthomonas* sp.	99.49	EF219047
M-M18	*Bacillus methylotrophicus*	99.86	KC790289
M-S1	*Pseudomonas alcaliphila*	99.92	CP016162
M-S2	*Lysinibacillus* sp.	98.6	JQ956507
M-S3	*Sphingomonas* sp.	99.63	KP866794
M-S4	*Acinetobacter junii*	99.85	KY767485
M-S5	*Bacillus* sp.	99.72	AM934692
M-S6	*Pseudomonas* sp.	99.78	KR063184
M-S7	*Acinetobacter* sp.	99.92	KX622562
M-S8	*Deinococcus* sp.	99.9	JQ511861
M-S9	*Janthinobacterium* sp.	98.09	KC207090
M-S10	*Acinetobacter modestus*	99.71	NR148845
M-S11	*Flavobacterium* sp.	99.45	AM177629
M-S12	*Fictibacillus* sp.	99.19	KX033807
M-S13	*Lysobacter* sp.	99.72	KT630892
M-S14	*Fluviicola* sp.	98.14	KY117481
M-S15	*Bacillus aryabhattai*	99.79	MF957305
M-S16	*Streptomyces* sp.	99.92	GQ395240
NPK-L1	*Bacillus cereus*	100	CP053954
NPK-L2	*Bacillus aryabhattai*	100	MT078622
NPK-L3	*Cupriavidus taiwanensis*	100	LT977002
NPK-L4	*Paenarthrobacter nitroguajacolicus*	100	KF555632
NPK-L5	*Bacillus velezensis*	100	MT081105

NPK-L6	*Pantoea agglomerans*	96.97	FJ357815
NPK-L7	*Bacillus megaterium*	100	KY621521
NPK-L8	*Bacillus safensis*	100	MT107116
NPK-L9	*Acinetobacter junii*	100	MN180840
NPK-L10	*Paenibacillus panacisoli*	99.65	KX827243
NPK-L11	*Paenibacillus xylanexedens*	100	KU239981
NPK-L12	*Clostridium tertium*	100	JX267105
NPK-L13	*Bacillus cereus*	99.78	MT020418
NPK-L14	*Stenotrophomonas* sp.	100	JQ917801
NPK-L15	*Bacillus safensis*	100	KX023225
NPK-L16	*Deinococcus* sp.	99.48	JQ511861
NPK-M1	*Stenotrophomonas maltophilia*	100	CP049956
NPK-M2	*Flavobacterium anhuiense*	99.34	JQ579648
NPK-M3	*Bacillus aryabhattai*	96.51	KJ009515
NPK-M4	*Bacillus* sp.	99.79	LC485468
NPK-M5	*Streptomyces tanashiensis*	98.86	MH497606
NPK-M6	*Acinetobacter junii*	100	MF101155
NPK-M7	*Bacillus lentus*	98.06	JX315557
NPK-M8	*Pseudomonas koreensis*	100	MT072188
NPK-M9	*Pseudomonas alcaligenes*	100	KC764976
NPK-M10	*Flavobacterium anhuiense*	100	JQ040003
NPK-M11	*Chryseobacterium* sp.	100	CP013293
NPK-M12	*Bacillus stratosphericus*	98	MT071728
NPK-S1	*Bacillus* sp.	98.89	MF139370
NPK-S2	*Acinetobacter junii*	100	MK053916
NPK-S3	*Arthrobacter* sp.	100	JN676108
NPK-S4	*Chryseobacterium* sp.	99.88	KY786115
NPK-S5	*Bacillus luciferensis*	100	JQ579634
NPK-S6	*Flavobacterium glaciei*	99.93	JF496402
NPK-S7	*Pseudomonas koreensis*	100	MT071422
NPK-S8	*Acinetobacter junii*	100	MF101155
NPK-S9	*Flavobacterium johnsoniae*	99.93	KU305720
NPK-S10	*Stenotrophomonas* sp.	100	MH883940
NPK-S11	*Comamonas testosteroni*	100	MF462924
NPK-S12	*Microbacterium hydrocarbonoxydans*	99.92	JF700471
NPK-S13	*Paenibacillus amylolyticus*	100	LN827736

Table S2 The 46 different single carbon resources used in this study.

Resource name	Resource classification
Sucrose	sugar
L-Glutamine	amino acid
Glycerol	other
Polysorbate	other
D-Mannose	sugar
Gelatin	other
Pectin	sugar
L-rhamnose	sugar
D-Fructose	sugar
D-Galactose	sugar
Citric acid	organic acid
Trehalose	sugar
Malic acid	organic acid
L-Arginine	amino acid
Propionic acid	organic acid
Stachyose	sugar
Maltose	sugar
Pyruvic acid	organic acid
Lactic acid	organic acid
Aspartic acid	amino acid
L-Phenyalanine	amino acid
Succinic acid	organic acid
L-Proline	organic acid
D-Ribose	sugar
Myoinositol	other
Ascorbic acid	organic acid
Creatinine	other
L-Serine	organic acid
Citrulline	amino acid
L-Threonine	organic acid
D-Xylose	sugar
D-Glutamine	amino acid
D-Serine	organic acid
Bromosuccinic acid	organic acid
L-Tryptophan	organic acid
L-Arabinose	sugar
Hydroxyacetic acid	organic acid
Quinic acid	organic acid
L-Leucine	organic acid
L-Lysine	organic acid
Isoleucine	organic acid
L-Histidine	amino acid
Maleic acid	organic acid
Mucus acid	organic acid
Pyroglutamic acid	organic acid
Melibiose	sugar
